# Feasibility of a Critical Care Ultrasound Curriculum Delivered Through Facebook

**DOI:** 10.7759/cureus.6349

**Published:** 2019-12-11

**Authors:** Shiqan Li, Alfredo Lee-Chang, Bassasm Yaghmour, Roozehra Khan, Janice Lieber, May M Lee

**Affiliations:** 1 Internal Medicine, Keck School of Medicine of the University of Southern California, Los Angeles, USA; 2 Pulmonary and Critical Care, Keck School of Medicine of the University of Southern California, Los Angeles, USA

**Keywords:** social media, facebook, critical care ultrasound, graduate medical education

## Abstract

Purpose

To investigate the feasibility of adjunct critical care ultrasound (CCUS) curriculum via Facebook, and evaluate its impact on fellow’s interest and knowledge acquisition.

Materials and methods

All University of Southern California (USC), Pulmonary, Critical Care and Sleep Medicine (PCCM) fellows were provided the usual CCUS curriculum. The intervention group provided access to an adjunct CCUS curriculum delivered via Facebook.

Results

Out of a total of 21 fellows, 10 (47.6%) participated in the Facebook group. The mean number of posts viewed was 24 with a range of 8 to 36 (total 41). Of those who responded, 56% responded Facebook was effective, 89% felt the content was moderate to very useful, 56% responded it enhanced their CCUS education, and 44% stated it motivated them to learn. Pre- and post-testing was done using paired t-tests; the average pre and post-intervention raw score means (of a total of 41 questions) for controls were 35.4±2.9 and 38.2±1.75 (p=0.005). Intervention scores were 37.56±1.94 and 38.0±1.50 (=0.602).

Conclusions

Evaluating the feasibility of the adjunct CCUS curriculum using social media, we found that Facebook may be acceptable to fellows, however, there was no significant improvement in knowledge. For learners, social media is easily accessible, widely available, and may motivate increased interest in learning and its potential uses warrants further study.

## Introduction

Critical care ultrasound (CCUS) has become recognized as an important skill for critical care physicians. Several national organizations have called for increased training and use of CCUS, emphasizing the importance of these skills. Although definitions of competencies have been developed, no consensus or standard approach to teaching critical care fellows CCUS skills is currently available [1-3]. 

In graduate medical education (GME) today, we are teaching a generation that has grown up with web-based media. Along with the increased availability of web-based resources, social media has become a ubiquitous part of daily life. Social media (e.g., Facebook, Twitter, Instagram, Snapchat, Reddit, etc.) has changed the way we communicate, obtain and disperse information, and interact. The education literature has shown that social media can increase engagement and interest in the subject matter being studied [4-6]. Because of its adaptable, customizable, and immediate nature, social media is compatible with several tenets of adult learning theory including relevancy of the material, active participation in the learning process and providing an informal and personal learning environment, and can potentially help with improved retention of material by a spaced learning approach [7-8]. Finally, social media is widely accessible with little to no financial costs to the page developer and user.

Unlike the use of social media, the use of internet-based learning is not new in teaching radiologic skills. Studies have shown that the integration of computer-based cases improves clinical students’ problem-solving ability in radiology and can be incorporated into the curriculum as a supplement to lectures [9-10]. However, it is not yet known whether social media can be used to enhance learning in a similar fashion. 

In this study, we investigated the feasibility of implementing a CCUS curriculum delivered via a social medial platform. There is literature to support social media use in education, however, it has not been studied in this setting. Technically, social media is relatively easy to use, both from a content creation standpoint and from a consumer standpoint. Economically, Facebook is a free tool. Presumed obstacles to the project included whether or not using a social medial platform for education would be acceptable to the fellows, and if the platform selected was the optimum medium. We hypothesized that by combining a web-based educational resource along with a convenient platform for delivery of content, using social media would lead to more interest and participation in continued learning outside the classroom setting. Additionally, we were interested in learning whether participation could lead to improved outcomes in the attitudes, skills, and knowledge of CCUS in pulmonary and critical care fellows.

## Materials and methods

This study was conducted within the pulmonary and critical care fellowship program at the University of Southern California (USC) Keck School of Medicine from September 2017 through June 2018. The Institutional Review Board at USC qualified this study as exempt from IRB review in August 2017.

**Figure 1:**
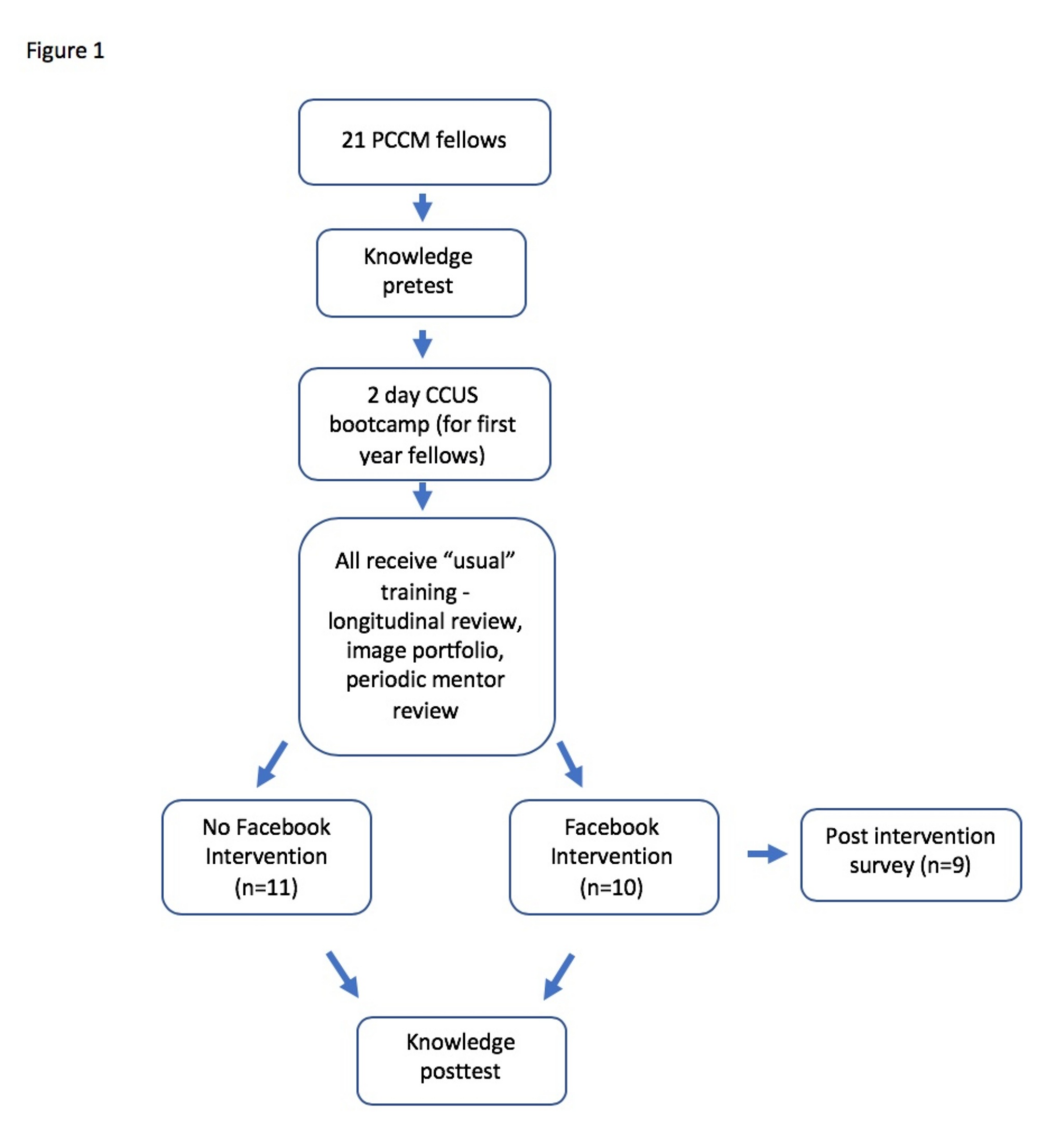
Study Design

We performed this prospective observational investigation of the effect of a CCUS curriculum delivered through the social media platform, Facebook (Figure 1). Our current CCUS curriculum consists of three distinct educational domains: basic ultrasonography and knobology, image acquisition skills, and image interpretation skills. There are two distinct phases of our CCUS training: (1) a two-day boot camp with traditional didactic teaching sessions, problem-based learning sessions that emphasize the clinical application of CCUS findings, and hands-on preceptor led training sessions; (2) a longitudinal review which emphasizes building an image portfolio with periodic mentor review.

In August 2017, prior to the boot camp, a pretest assessment of skills and knowledge was performed on all 21 of the pulmonary and critical care fellows using a knowledge assessment tool with permission from the authors [11]. In September 2017, the boot camp was provided to the first-year fellows. Senior-level fellows had prior exposure to the boot camp in previous years. A Facebook “Critical Care Ultrasound Forum” was then created in October 2017 and fellows were given the option to voluntarily join this group. This ultrasound forum consisted of approximately 40 core skills divided into five systems - basic ultrasound, pulmonary ultrasound, cardiac ultrasound, abdominal ultrasound and vascular ultrasound deployed over four months with one-to-two posts per week. The content was presented on Facebook as quizzes, cases, or management-type questions along with links to webpages, journal articles and other supplemental information for those who wished to learn more about the specific topic. Towards the end of the academic year, we concluded our forum and downloaded data from Facebook looking at page traffic, number of views and usage over time. At the conclusion of the forum, we retested all fellows with the same knowledge assessment and also distributed a 14 question post-forum survey to the fellows that participated gauging interest, accessibility, and effectiveness of the platform as a whole as a means of delivering CCUS education.

Statistical analysis was performed using Statistical Package for the Social Sciences (SPSS Inc., Chicago, IL) to perform paired t-tests to compare pre- and post-test results and significance viewed as p<0.05.

## Results

Demographics

Out of a total of 21 fellows, there were 10 (48%) that participated in the Facebook group, which included four first-year, four second-year, and two third-year fellows. Table 1 describes the study participants.

**Table 1:** Study Participants

Parameter	N	Percent
Level of Training		
Fellow Year 1 (total 7)	4	57.1
Fellow Year 2 (total 7)	4	57.1
Fellow Year 3 (total 7)	2	28.6
Gender		
Male (total 15)	7	46.7
Female (total 6)	3	50.0

Usage

**Figure 2:**
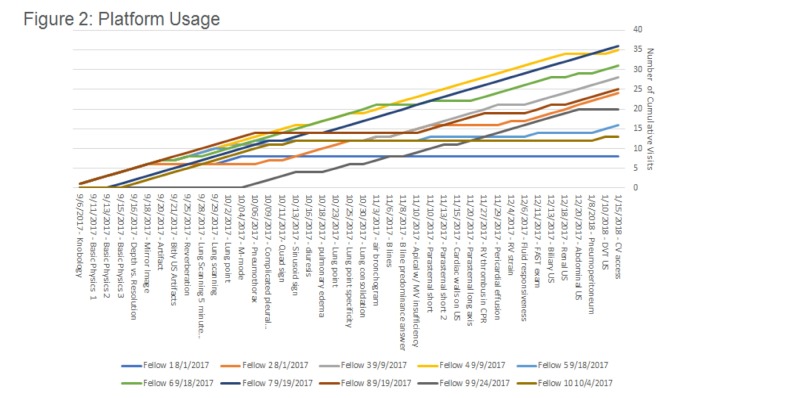
Platform Usage

Forty-one total posts were generated in the Facebook group with the mean number of posts viewed being 24 with a range from 8 to 36. As shown in Figure 2, the majority of those that participated continued to follow along, as demonstrated by continued positive slopes throughout the forum. 

Survey

Of the 10 distributed, there were nine responses (90%) to the post-intervention survey with 44% responding that they would participate again in a Facebook education group and 33% responding maybe. 56% responded that Facebook was an effective platform for delivering content. 89% responded that the content was moderate to very useful. 56% responded that it enhanced their CCUS education with 44% stating it motivated them to learn more.

Pre and post testing

Both a pre- and post-intervention test were given to all the fellows regardless of participation in the Facebook curriculum with a perfect score being 41 correct. One pre-intervention test was started but not completed by a participant, so the pre and post-test data for this fellow was omitted from the analysis. The average overall pre and post-test mean scores were compared using paired t-tests. Raw test score (of a total of 41) results divided by fellowship year were: first years 34.67 ± 2.66 and 37.33 ± 0.8, p=0.025, n=6; second years 37.0 ± 3.46 and 37.86 ± 1.68, p=0.57, n= 7; third years 36.43 ± 2.64 and 38.86 ± 1.77, p=0.31. n= 7 (Table 2). The average pre-intervention means for those who did not participate (control) and those who did (intervention) were 35.4 ± 2.9 and 37.56 ± 1.94, p=0.005 with post-interventions mean of 38.2±1.75 and 39.0±1.50, p=0.602, respectively (Table 3).

**Table 2:** Comparison of Means Amongst Fellows *highest raw score is 41

Fellow Year	Raw Pretest Means* (SD)	Prestest % correct	Raw posttest Means (SD)	Posttest % correct	p-value
First year (n=6)	34.67 (2.66)	84.56	37.33 (0.82)	91.05	0.025
Second Year (n=7)	37.00 (3.46)	90.20	37.86 (1.68)	92.34	0.573
Third Year (n=7)	36.43 (2.64)	88.85	38.86 (1.77)	94.78	0.031
All three years combined	36.10 (2.97)	88.04	38.05 (1.57)	92.80	0.006

**Table 3:** Comparison of Test Means Comparing Control vs Facebook Intervention Groups *highest raw score is 41

Group	Raw Pretest Means* (SD)	Prestest % correct	Raw posttest Means (SD)	Posttest % correct	p-value
All years control (n=11)	35.40 (2.91)	86.34	38.20 (1.75)	93.17	0.005
All years intervention (n=9)	37.56 (1.94)	91.61	38.00 (1.50)	92.68	0.602
First Years control (n=3)	33.00 (1.73)	80.49	37.00 (1.00)	90.24	0.020
First years Intervention (n=3)	36.33 (2.52)	88.61	37.70 (0.58)	91.95	0.383

## Discussion

Our principal goal in this study was to determine the feasibility of implementing a CCUS curriculum through social media. We do believe that a CCUS curriculum can be successfully accomplished using social media, as demonstrated by the ease of creation of the forum, ease of access, level of participation, and the overall positive feedback received from those that participated. Content creation for the 40 core skills was divided amongst pulmonary and critical care faculty and easily presented through Facebook’s accessible and widespread medium. 

Access was readily available and accessible to all those that voluntarily chose to participate. Voluntary participation, rather than mandatory, in our intervention group was chosen for at least two practical reasons. We wished to respect our fellow’s limited time and engage only those who were interested. Additionally, we want to use Facebook as an adjunct to-and not a substitute for-our more traditional CCUS teaching methods. Along with Facebook, several social media platforms were considered for this study: Instagram, Twitter, and Viber. Each platform has its benefits and limitations. We chose Facebook for several reasons. First, according to the Pew Research foundation in 2018, Facebook remains the most popular social media platform and most of our trainees already have accounts [12]. Second, Facebook allows the creation of private, invitation-only pages where we can limit the distribution of our content to only our target audience. Third, it allows for the posting of images, videos, quizzes, and text as well as links to outside sources, which, as described below, was important for our study. Finally, data can be downloaded directly from the platform, which facilitates analysis of the results of the intervention. The information available includes the number of likes, number of engaged users, total reach, and others.

Overall, the mean test scores increased in both Facebook and non-Facebook groups. However, it was notable that there was a significant improvement of the means in the non-Facebook group when compared to the Facebook group. We hypothesize a couple of different factors for this surprising finding. The first factor may have been that those who chose to participate in the Facebook group had higher pre-test scores compared with those who did not join. This self-selected Facebook training group may have had more interest in CCUS, to begin with, possibly reflected through their initial scores. This could also suggest that there may have been less knowledge overall to gain from the course compared to those in the non-Facebook group. This is demonstrated by a subgroup analysis of the fellowship years, with only the first years and the third years continued to have a significant negative correlation comparing Facebook and non-Facebook groups. The first-year fellows may have had the self-selection bias, and the third-year fellows have had extra years of training and would most likely have less to learn than the others. Additionally, our pre- and post-test instrument may not have been sensitive enough to detect true differences in knowledge, as indicated by the elevated mean pre-test scores of >85% for all the fellows. Lastly, although 21 fellows are a relatively large fellowship program, our subject size still remains limited and is reflected by the narrow confidence intervals in our comparisons which reflect that our true mean difference is smaller despite the significant p-value. 

Among a heterogeneous group of fellows at different years of training, the overall responses from the post-intervention survey were positive. Most of the fellows that participated would join a similar group again if given the choice again in the future and a small majority believed that the medium was an effective learning tool. 

In the future, mandatory participation could increase the number of those participating in the study and would remove the self-selection aspect of the study. Given that the confidence intervals were narrow, increasing the study size would help to power our study further and would be done in future studies. Asking those that may not have self-selected whether this endeavor was worthwhile may be a bonus in assessing the utility of social media in education. Additionally, we chose Facebook for the reasons mentioned above. We could consider a different social media platform, such as Instagram, to deliver content as suggested by the fellows. In the post survey, there were indications that a different platform may have been more engaging with fellows, with many suggesting the use of Instagram rather than Facebook. Lastly, we could consider using a sample more naive to CCUS to test our hypothesis or use a more discerning pre and posttest to assess knowledge.

## Conclusions

In conclusion, despite our negative test results with regard to knowledge improvement, we feel that social media may be a feasible way to augment a CCUS program, given its wide acceptability by trainees. It is easily accessible, widely available, free to use, has a potential broad reach, and can motivate learners and increase interest in learning CCUS.
